# mGEM: multigraph estimation models for pattern analysis

**DOI:** 10.1186/s12859-026-06467-3

**Published:** 2026-05-20

**Authors:** Alfonso Landeros, Dhwani Krishnan, Kenneth Lange, Mary Sehl

**Affiliations:** 1 Department of Computational Medicine, David Geffen School of Medicine at UCLA, Los Angeles, CA 90095-1554 USA; 2 Department of Human Genetics, David Geffen School of Medicine at UCLA, Los Angeles, CA 90095-1554 USA; 3 Department of Statistics, College of Letters and Science at UCLA, Los Angeles, CA 90095-1554 USA; 4 Division of Hematology-Oncology, David Geffen School of Medicine at UCLA, Los Angeles, CA 90095-1554 USA; 5 Department of Statistics, College of Natural and Agricultural Sciences at UCR, Riverside, CA 9251-0001 USA; 6 Wayne State University School of Medicine, Detroit, MI 48201 USA

**Keywords:** Co-occurrence analysis, Multigraphs, Count models, Co-expression networks, Regression

## Abstract

**Background:**

Many networks contain node and edge data in the form of node-specific covariates and edge weights, respectively. Established methods for investigating graph structure often rely on dichotimizing edge data. This simplification motivates the development of techniques for multivariate analysis. In the context of biological networks, such as in gene co-expression analysis, flexible tools and models are needed to interrogate data for enrichment and differential interactions. We present Multigraph Estimation Models (mGEM) as flexible alternatives to correlation-based methods for analyzing co-occurrence and co-expression data.

**Methods:**

The mGEM approach interprets non-negative integer-valued edge weights as multiple edges in a multigraph and models the expected number of edges between nodes through a propensity-based relation. Overdispersion and node-specific attributes are treated under a unified parametric framework by changing distributional assumptions and reparameterization, respectively. By providing a background model for comparison, we are able to compute residuals that, in turn, are used to rank associations between nodes using mutual ranks and identify dependent components.

**Conclusions:**

We consider several simulated and real data examples including a classic nematode neural network, a manually-added co-authorship network derived from Google Scholar, and *Saccharomyces cerevisiae* co-expression data from RNA-seq experiments. These examples illustrate how our models can be used in exploratory data analysis to generate potentially interesting hypotheses. For example, our co-authorship analysis extracts known collaborations between universities and reveals connections between fields of interest. In analyzing co-expression data, mGEM posits several gene modules that share similarities with correlation-based analyses yet exhibit greater diversity in terms of co-expression propensities.

## Background

The last few decades have seen considerable development in applied network theory. Exponential Random Graph Models (ERGM) [1, 2] and the closely related class of stochastic block models [3, 4] have important applications to networks from a broad variety of fields, including physics, chemistry, biology, ecology, sociology, and information science. Biological examples feature gene regulation networks and neural networks. Information science stresses co-author networks. The literature on ERGMs typically emphasizes tie analysis that treats edges (dyads) as 0-1 random variables to interrogate relationships between nodes (actors). Recent work has shifted attention towards a multivariate perspective, such as in *valued networks* [5, 6], *weighted stochastic block models* [7], and *random multigraphs* [8, 9]. These developments extend network analysis to incorporate both quantitative and qualitative node and edge data. To capture close associations in the multivariate setting, connections between nodes are either weighted or interpreted as repeated interactions. We adopt the latter perspective using random multigraphs.

Briefly, multigraphs consist of nodes and a random number of edges connecting each pair of nodes. Propensities, two for each pair of nodes or one from each node to the other in a directed graph, quantify the tendency of nodes to form edges and can be estimated using maximum likelihood algorithms. Given a parametric model and estimates of node propensities under the model, one can then compare observed to expected numbers of edges and test for associations between nodes. It is known that propensity-based random multigraph models do not capture clustering effects or other phenomena observed in some classes of networks, such as scale-free or preferential attachment networks. Ranola et al. developed a modification of Poisson random multigraphs that accounts for clustering, the clustering and propensity based approximation (CPBA), and showed that CPBA is a sparse approximation to adjacency matrices [10]. The CPBA is also established as a generalization of correlation network analysis.

### Our contributions

In the current article, we expand on the *undirected* random Poisson multigraph model [8] and the modeling ideas that motivated it. We begin by first deriving a random multigraph model based on the negative binomial distribution. Next, we take a high-level view of the propensity-based models described by Ranola et al. and propose a generalization incorporating node-specific attributes as covariates via regression. In each setting, we present practical MM algorithms [11] estimating model parameters, including both propensities and regression coefficients, under a maximum likelihood framework. Various simulation studies illustrate the merits and limitations of our models. Our algorithms are implemented in the multi-threaded Julia software MultigraphEstimationModels.jl, which we abbreviate as mGEM. It is distributed as free open-source software on GitHub, https://github.com/alanderos91/MultigraphEstimationModels, under the MIT “Expat” license.

We apply our random multigraph theory to analyze data from three separate networks, highlighting the broad range of datasets mGEM can be applied to. We begin with a molecular dataset, specifically to analyze gene counts from RNA-seq experiments in *Saccharomyces cerevisiae* [12]. After fitting an mGEM to co-expression data, we compute and rank residuals, defined as the differences between observed and expected co-expression counts. Groups of associated genes are extracted from the connected components of induced undirected graphs derived from model residuals. Our analysis illustrates how mGEM can be used as an exploratory tool, in conjunction with enrichment analysis, to extract meaning from co-expression patterns and generate hypotheses for further consideration. We then proceed at the cellular level to analyze the classic *Caenorhabditis elegans* neural network [13]. Comparing results, we find reasonably matching estimates of mean number of edges between pairs of neurons under both the Poisson and negative binomial formulations of mGEM. Most associations are stable across the two distributional assumptions, matching previously reported results [8]. Finally, to demonstrate the applicability of mGEM to non-biological data, we also apply our random multigraph theory to analyze network data derived from Google Scholar citation counts for pairs of authors [14]. We examine factors driving associations between fields of interest, including gender and bibliometrics. These findings demonstrate how to incorporate information on node-specific covariates into a framework resembling generalized linear models. Ranking residuals reveals relationships between authors’ affiliated institutes and fields of interest. In summary, our examples demonstrate how mGEM can be adapted to handle various situations encountered in both biological and non-biological applied data analysis.

Before proceeding, we note that the *undirected* assumption is not strictly necessary. We focus on this case for the sake of presentation and note that all our models and methods have generalizations to the directed graph case [8].

## Methods

### Overview of data, models, and estimation procedures

Abstractly, we consider non-negative, integer data in symmetric $n \times n$ matrix form, $\boldsymbol{Z}= (Z_{ij})$, which define a multigraph on *n* nodes. The entries $Z_{ij} \ge 0$ indicate the number of edges connecting two distinct nodes, *i* and *j*. We only consider undirected multigraphs so that $Z_{ij} = Z_{ji}$. This symmetry will be invoked in some of the derivations that follow but it is not essential. Our theory excludes self-edges, and we emphasize this exclusion by adopting the convenient convention $Z_{ii} = 0$ for each node *i*.

In practice, the integers $Z_{ij}$ count the completely observed number of interactions between two entities or classes of entities, labeled as *i* and *j*. Our theory is developed under the critical assumption of independent edge formation, which means that node *i* does not influence the tendency of node *j* to form edges. This translates to $Z_{ij}$ being independent of $Z_{k\ell }$ in modeling the data with random variables. The entities modeled by nodes on a multigraph are otherwise allowed to interact. In practice, there is nuance in whether one is assuming independence on nodes (the entities observed) or on multi-edges (interactions between the entities). Independent edge formation may not hold in some applications, yet it is neither an issue for analysis nor a restrictive assumption. Our primary goal is to develop a null model for analyzing patterns encoded by co-occurrence data $\boldsymbol{Z}$, which can used to identify associations by examining deviations from the expected statistics predicted by it. Stated explicitly, our methods are used to propose potentially interesting associations rather than detect or recover structure in a network. For example, the co-expression data example in Sect. "Gene co-expression in *Saccharomyces cerevisiae*" considers counts $Z_{ij}$ tracking the number of times different animals had co-expression events for genes *i* and *j* and likely satisfies independent edge formation. In contrast, the co-authorship data example in Sect. "Google Scholar co-authorship analysis" uncovers intuitive associations between research institutions even though such relationships are likely not formed independently of each other. In our view, incorporating prior knowledge into a random multigraph model is a separate issue that merits careful consideration in future work.

Having clarified the structure of the data under consideration, we now briefly introduce our model framework. Assuming *n* nodes form edges in an independent fashion, we formulate likelihood models

$$\begin{aligned} \mathcal {L}(\boldsymbol{\theta }\mid \boldsymbol{Z}) = \prod _{\{i,j\}} \Pr (Z_{ij} = z_{ij}) = \prod _{i=1}^{n} \prod _{j\ne i}^{n}\Pr (Z_{ij} = z_{ij}) \end{aligned}$$

to estimate parameters of interest, $\boldsymbol{\theta }$, via maximum likelihood estimation. The generic index notation $\{i,j\}$ in the first equality enumerates all admissible pairs of nodes on the multigraph encoded by $\boldsymbol{Z}$. Here $\boldsymbol{\theta }$ denotes a finite, generic parameter vector that concatenates propensities $\boldsymbol{p}= (p_{j})$ and any nuisance parameters, such as the scale parameter *r* in the negative binomial distribution. In models that relate propensities to node-specific covariates, one substitutes attribute coefficients $\boldsymbol{\beta }= (\beta _{j})$ for propensities in the parameter vector $\boldsymbol{\theta }$. In the forthcoming section, we relate component-wise expectations of the $n \times n$ data $\boldsymbol{Z}$ to the *n* propensity parameters $\boldsymbol{p}$; denote these quantities by the notation $\boldsymbol{\mu }\equiv \mathrm{E\,\!}[\boldsymbol{Z}] = (\mathrm{E\,\!}[Z_{ij}])$.

Because the models considered do not have analytic solutions, we invoke the minorization-maximization (MM) principle to derive iterative algorithms that reliably recover solution estimates [11]. Briefly, the MM principle substitutes an objective *f*(*x*), which we seek to maximize, with a suitably chosen surrogate model $g(x \mid y)$ anchored at a point *y*. In our applications the surrogate model $g(x \mid y)$ is chosen through clever application of mathematical inequalities to separate parameters that are otherwise coupled in the objective *f*(*x*). A surrogate is often denoted $g(x \mid x_{m})$ to suggest it is anchored at the previous iterate $x_{m}$. The next iterate $x_{m+1}$ is calculated by solving a subproblem $x_{m+1} = \mathrm {arg~max}_{x} g(x \mid x_{m})$. By construction, iterates chosen via the MM principle automatically satisfy the ascent condition, $f(x_{m}) \le f(x_{m+1})$.

### Propensity-based multigraph models

In propensity models, the probability of observing *z* edge counts between nodes *i* and *j*, the event $\{Z_{ij} = z\}$, is

1 $$\begin{aligned} \Pr (Z_{ij} = z) = f(z \mid p_{i}, p_{j}, \theta _{1}, \theta _{2}, \ldots \theta _{K}); \qquad \text {for}~i,j=1,2,\ldots ,n \end{aligned}$$

where $f(z, \mid p_{i}, p_{j}, \theta _{1}, \theta _{2}, \ldots , \theta _{K})$ is a probability mass function that depends on propensities $p_{i}$ and $p_{j}$ and finitely many additional parameters $\theta _{1}, \theta _{2}, \ldots , \theta _{K}$ that are *not* node-specific. The non-negative parameters $p_{i}$ are deemed propensities because they quantify the tendency that node *i* forms an edge with another node. The functional form of probabilities given in Eq. (1) is fixed across node pairs so that node propensities drive variation in edge counts. Specifically, we model the mean of $Z_{ij}$ by taking

2 $$\begin{aligned} \mu _{ij} \equiv \mathrm{E\,\!}[Z_{ij}] = p_{i} \cdot p_{j}, \end{aligned}$$

under the assumption of independent edge formation and restrict the random edge counts $Z_{ij}$ to a particular family of distributions.

#### Poisson edges

Assuming edge counts $Z_{ij}$ obey a Poisson distribution, the model specified by Eqs. (1) and (2) yield the log-likelihood

3 $$\begin{aligned} \mathop {\ln \mathcal {L}}\nolimits (\boldsymbol{p}\mid \boldsymbol{Z}) = \sum _{\{i,j\}} \left[ Z_{ij} \ln \mu _{ij} - \mu _{ij} - (Z_{ij})! \right] = \sum _{\{i,j\}} \left[ Z_{ij} (\ln p_{i} + \ln p_{j}) - p_{i} p_{j} - (Z_{ij})! \right] , \end{aligned}$$

in the absence of covariates, where the sum is taken over pairs (*i*, *j*) such that $i \ne j$. We apply the majorization $xy \le \frac{y_{m}}{2x_{m}}x^2 + \frac{x_{m}}{2y_{m}}y^2$ to log-likelihood (3) to derive the useful surrogate

$$\begin{aligned} g(\boldsymbol{p}\mid \boldsymbol{p}_{m}) = \sum _{\{i,j\}} \left[ Z_{ij} (\ln p_{i} + \ln p_{j}) - \frac{p_{m,j}}{2 p_{m,i}} p_{i}^{2} - \frac{p_{m,i}}{2 p_{m,j}} p_{j}^{2} - (Z_{ij})! \right] , \end{aligned}$$

which conveniently splits parameters $p_{i}$ and $p_{j}$ [8]. Differentiating with respect to $p_{i}$ and setting derivatives equal to 0 results in a quadratic equation in the variable $p_{i}$. This result defines the MM updates

4 $$\begin{aligned} p_{m+1,i} = \sqrt{ \frac{p_{m,i} \sum _{j \ne i} Z_{ij}}{\sum _{j \ne i} p_{m,j}} },~\text {for}~i=1,2,\ldots ,n. \end{aligned}$$

The iterative map (4) respects non-negativity constraints on propensities, can be applied in parallel to each propensity *i*, and is a continuous, one-to-one function of the sufficient statistics $T_{i}(\boldsymbol{Z}) = \sum _{j \ne i} Z_{ij}$. Iterating the map recovers the unique maximum likelihood estimate of node propensities [8].

#### Negative binomial edges

For over- or under-dispersed data, assuming a negative binomial distribution on edge counts may be more appropriate than the Poisson model described above. The negative binomial distribution, denoted $\textrm{NB}(r,\pi )$, has probability mass function

$$\begin{aligned} \Pr (Z = z) = \frac{\Gamma (z+r)}{\Gamma (z+1) \cdot \Gamma (r)} \pi ^{z} (1-\pi )^{r}; \qquad \text {where}~r > 0~\text {and}~\pi \in [0,1]. \end{aligned}$$

We can interpret *z* as the number of successes in independent Bernoulli trials with probability $\pi$ until one observes *r* failures. To match the propensity formulation of our multigraph models in Eq. (1), it is convenient to reparameterize the distribution in terms of $\mu _{ij} \equiv \mathrm{E\,\!}[Z_{ij}]$. Setting $\pi _{ij} = \mu _{ij}(\mu _{ij}+r)^{-1}$ and taking $r \rightarrow \infty$ shows that $\textrm{NB}(r, \pi _{ij})$ converges to a Poisson distribution $\textrm{Poisson}(\mu _{ij})$. In addition to matching Eq. (1), this perspective emphasizes the mean-variance relationship, $\textrm{Var}[Z_{ij}] = \mu _{ij} + r\mu _{ij}^{2}$, between the Poisson and negative binomial distributions. Alternatively, we may parameterize the probability model as $\textrm{NB}(\alpha ,\pi _{ij})$, where $\alpha = 1/r$, to improve on numerical estimation of *r* but omit this direction from the present work. We propose a block coordinate ascent algorithm to fit maximum likelihood estimates to data. Namely, we alternate between updates of $\boldsymbol{p}$ for fixed *r* and vice-versa in an iterative fashion. The *supporting hyperplane inequality*

$$\begin{aligned} f(x) \ge f(y) + df(y)(x - y), \end{aligned}$$

for a convex function *f*(*x*), with differential *df*(*x*), plays central role in our derivations.

Under the $\textrm{NB}(r, \pi _{ij})$ parameterization of the negative binomial, the log-likelihood of interest is

5 $$\begin{aligned} \ln \mathcal {L}(\boldsymbol{p},r) = \sum _{\{i,j\}} \left\{ Z_{ij} \ln \pi _{ij} + r \ln (1-\pi _{ij}) - \left[ \ln (Z_{ij} + r) + \ln B(Z_{ij}+1,r) \right] \right\} , \end{aligned}$$

where $\pi _{ij} = \mu _{ij} / (\mu _{ij} + r)$ and $\mu _{ij} = p_{i} p_{j}$. Here *B*(*x*, *y*) denotes the beta function with arguments *x* and *y*,

$$\begin{aligned} B(x,y) = \int _{0}^{1} t^{x-1}(1-t)^{y-1}~\textrm{d}t. \end{aligned}$$

We first fix *r* and view the log-likelihood (5) as a function of $\boldsymbol{\mu }$. Ignoring terms $c_{m,ij}$ which are constant with respect to $\boldsymbol{\mu }$, the supporting hyperplane inequality suggests the surrogate

$$\begin{aligned} g(\boldsymbol{\mu }\mid \boldsymbol{\mu }_{m}) = \sum _{\{i,j\}}&Z_{ij} \ln \mu _{ij} - \frac{Z_{ij}+r}{\mu _{m,ij}+r}(\mu _{ij}-\mu _{m,ij}) + c_{m,ij}, \end{aligned}$$

minorizing $\ln \mathcal {L}(\boldsymbol{\mu }\mid r)$ and separating $\boldsymbol{\mu }$ and *r*. The chain rule reveals the derivative of $g(\boldsymbol{\mu }\mid \boldsymbol{\mu }_{m})$ with respect to $p_{k}$

$$\begin{aligned} \sum _{j \ne k} Z_{kj} \frac{1}{\mu _{kj}} \cdot \frac{\partial \mu _{kj}}{\partial p_{k}} - \frac{Z_{kj}+r}{\mu _{m,kj}+r} \cdot \frac{\partial \mu _{kj}}{\partial p_{k}}, \quad \text {where} \quad \frac{\partial \mu _{kj}}{\partial p_{k}} = p_{j}. \end{aligned}$$

Setting this equal to 0 and solving for $p_{k}$ yields the propensity update

6 $$\begin{aligned} p_{m+1,i} = \frac{ \sum _{j \ne i} Z_{ij} }{ \sum _{j \ne i} \frac{Z_{ij} + r}{\mu _{m,ij} + r} \cdot p_{m,j}}, \qquad \text {for}~i=1,2,\ldots ,n. \end{aligned}$$

Now we fix $\boldsymbol{p}$ and derive a surrogate to aid in updating *r*. The identity $\ln [\Gamma (x+r)/\Gamma (r)] = \sum _{k=0}^{x-1} \ln (r+k)$ for integer-valued *x* and the supporting hyperplane inequality, applied term-by-term to logarithms, lead to the minorizing surrogate

$$\begin{aligned} g(r \mid r_{m}) = \sum _{\{i,j\}} \left[ \sum _{k=0}^{Z_{ij}-1} \frac{r_{m}}{r_{m}+k}\ln r + \left[ \ln (1-\pi _{m,ij}) + \pi _{m,ij} - Z_{ij}(1-\pi _{m,ij})r_{m}^{-1}\right] (r-r_{m}) + c_{m,ij} \right] . \end{aligned}$$

Only terms involving $\ln r$ and *r* are pertinent to maximization; all other items are collected in the constant $c_{m,ij}$. We differentiate with respect to *r* and solve the stationary condition $g(r\mid r_{m}) = 0$ to identify the *r* update:

7 $$\begin{aligned} r_{m+1} = \frac{ -\sum _{\{i,j\}} \sum _{k=0}^{Z_{ij}-1} \frac{r_{m}}{r_{m}+k} }{ \sum _{\{i,j\}} \ln (1-\pi _{m,ij}) + \frac{\mu _{m,ij} - Z_{ij}}{\mu _{m,ij} + r_{m}} }. \end{aligned}$$

Compared to Newton’s method, the update in (7) guarantees ascent without backtracking or line searches.

### Covariate-based multigraph models

The propensity model in Eq. (1) can be expanded to incorporate node attributes as covariates influencing a node’s propensity for edge formation. For example, given a set of *d* features denoted $\boldsymbol{x}_{i} = (x_{i1}, x_{i2}, \ldots x_{id}) \in \mathbb {R}^{d}$, we model the propensity of node *i* as

$$\begin{aligned} \mathrm{E\,\!}[p_{i} \mid \boldsymbol{x}_{i}] = g\left( \sum _{k=1}^{d} x_{ik} \beta _{k} + \beta _{i0} \right) = g(\boldsymbol{x}_{i}^{\top } \boldsymbol{\beta }+ \beta _{i0}). \end{aligned}$$

The coefficient vector $\boldsymbol{\beta }= (\beta _{k}) \in \mathbb {R}^{d}$ captures the effect of each covariate and $g(\cdot )$ acts as an inverse link relating the mean effect $\boldsymbol{x}_{i}^{\top } \boldsymbol{\beta }$ to its corresponding node propensity $p_{i}$. Note that $\boldsymbol{\beta }$ is shared across all nodes whereas the intercept terms $\beta _{i0}$ are specific to each node *i*. Ideally, $g(\cdot )$ should be continuous, monotonic, and non-negative so as to satisfy the requirement $p_{i} > 0$ and adequately capture the meaning of $p_{i}$ as a propensity. A good candidate is the exponential function $g(x) = e^{x}$ which we focus on for the remainder of this paper. In summary, the covariate model is specified by

8 $$\begin{aligned} \begin{aligned} \mathrm{E\,\!}[p_{i} \mid \boldsymbol{x}_{i}]&= g(\boldsymbol{x}_{i}^{\top } \boldsymbol{\beta }) = e^{\boldsymbol{x}_{i}^{\top } \boldsymbol{\beta }} \\ \Pr (Z_{ij} = z \mid \boldsymbol{X})&= f(z \mid p_{i}, p_{j}, \boldsymbol{x}_{i}, \boldsymbol{x}_{j}) \\ \mathrm{E\,\!}[Z_{ij} \mid \boldsymbol{X}]&= \mu _{ij}(\boldsymbol{\beta }) = g(\boldsymbol{x}_{i}^{\top }\boldsymbol{\beta }) \cdot g(\boldsymbol{x}_{j}^{\top }\boldsymbol{\beta }) \quad \text {for}~i,j=1,2,\ldots ,n \end{aligned} \end{aligned}$$

Note that our choice of inverse link sets each mean $\mu _{ij}(\boldsymbol{\beta }) = \exp \{(\boldsymbol{x}_{i} + \boldsymbol{x}_{j})^{\top } \boldsymbol{\beta }\}$. Thus, nodes with similar attributes tend to form edges when their mean effects $\boldsymbol{x}_{i}^{\top }\boldsymbol{\beta }$ and $\boldsymbol{x}_{j}^{\top }\boldsymbol{\beta }$ both have positive signs, or when one node’s positive mean effect dominates the other. This is consistent with the propensity model (1). The advantage of the covariate model is that it allows for additional analysis provided covariates are chosen appropriately. In addition, passing from node propensities to node covariates can dramatically reduce the number of model parameters. Implicit in model (8) is the assumption that nodes are homogeneous in some sense.

In the covariate setting, our strategy for maximum likelihood estimation is to iteratively estimate model coefficients $\boldsymbol{\beta }$ via a Fisher scoring algorithm

9 $$\begin{aligned} \boldsymbol{\beta }_{m+1} = \boldsymbol{\beta }_{m} + t_{m} \mathcal {I}^{-1}(\boldsymbol{\beta }_{m})~\nabla _{\boldsymbol{\beta }_{m}}\mathop {\ln \mathcal {L}}\nolimits (\boldsymbol{\beta }_{m} \mid \boldsymbol{Z}), \end{aligned}$$

where $t_{m} \in (0,1]$ is a step size chosen via backtracking and $\mathcal {I}(\boldsymbol{\beta }) = \mathrm{E\,\!}[-\nabla _{\boldsymbol{\beta }}^{2}\mathop {\ln \mathcal {L}}\nolimits (\boldsymbol{\beta }\mid \boldsymbol{Z})]$ denotes the expected information matrix. We now turn our attention to the specific cases of Poisson and negative binomial edges.

#### Poisson edges

Assuming the independent counts $Z_{ij}$ are Poissonian, $Z_{ij} \sim \textrm{Poisson}(\mu _{ij})$, our log-likelihood model takes the form

10 $$\begin{aligned} \mathop {\ln \mathcal {L}}\nolimits (\boldsymbol{\beta }\mid \boldsymbol{Z}) = \sum _{\{i,j\}} \left[ Z_{ij} \ln \mu _{ij}(\boldsymbol{\beta }) - \mu _{ij}(\boldsymbol{\beta }) - (Z_{ij})! \right] = \sum _{\{i,j\}}\left[ Z_{ij} (\boldsymbol{x}_{i} + \boldsymbol{x}_{j})^{\top }\boldsymbol{\beta }- e^{(\boldsymbol{x}_{i} + \boldsymbol{x}_{j})^{\top }\boldsymbol{\beta }} - (Z_{ij})! \right] . \end{aligned}$$

Ignoring terms independent of $\boldsymbol{\beta }$, it is clear the log-likelihood is a linear combination of affine and concave terms and hence is concave in $\boldsymbol{\beta }$. Invoking symmetry of the undirected graph, elementary calculus establishes that the gradient and Hessian of the Poisson model (10) with respect to coefficients $\boldsymbol{\beta }$ are

11 $$\begin{aligned} \begin{aligned} \nabla _{\boldsymbol{\beta }} \mathop {\ln \mathcal {L}}\nolimits (\boldsymbol{\beta }\mid \boldsymbol{Z})&= \sum _{\{i,j\}}~[Z_{ij} - \mu _{ij}(\boldsymbol{\beta })]~(\boldsymbol{x}_{i}+\boldsymbol{x}_{j}) = 2 \boldsymbol{X}(\boldsymbol{Z}- \boldsymbol{\mu }) \textbf{1}, \\ \nabla _{\boldsymbol{\beta }}^{2} \mathop {\ln \mathcal {L}}\nolimits (\boldsymbol{\beta }\mid \boldsymbol{Z})&= -\sum _{\{i,j\}}~\mu _{ij}(\boldsymbol{\beta }) \cdot (\boldsymbol{x}_{i}+\boldsymbol{x}_{j})~(\boldsymbol{x}_{i}+\boldsymbol{x}_{j})^{\top } = -2 \boldsymbol{X}[\textrm{Diag}(\boldsymbol{\mu }\textbf{1}) + \boldsymbol{\mu }]\boldsymbol{X}^{\top }. \end{aligned} \end{aligned}$$

Here $\boldsymbol{X}$ is a $d \times n$ matrix formed by horizontal concatenation of the *n* feature vectors $\boldsymbol{x}_{i} \in \mathbb {R}^{d}$ for each node *i*. Note that computing the expected information matrix $\mathcal {I}(\boldsymbol{\beta })$ merely changes signs in the Hessian $\nabla _{\boldsymbol{\beta }}^{2}\mathop {\ln \mathcal {L}}\nolimits (\boldsymbol{\beta }\mid \boldsymbol{Z})$.

#### Negative binomial edges

When the independent counts $Z_{ij}$ obey negative binomial distributions, $Z_{ij} \sim \textrm{NB}(r,\pi _{ij})$ with $\pi _{ij} = \mu _{ij} (\mu _{ij} + r)^{-1}$, careful application of the chain rule to (5), the equation $\nabla _{\boldsymbol{\beta }} \pi _{ij}(\boldsymbol{\beta }) = \pi _{ij}(1-\pi _{ij})(\boldsymbol{x}_{i} + \boldsymbol{x}_{j})$, and symmetry yield

12 $$\begin{aligned} \begin{aligned} \nabla _{\boldsymbol{\beta }} \mathop {\ln \mathcal {L}}\nolimits (\boldsymbol{\beta }\mid \boldsymbol{Z})&= \sum _{\{i,j\}} \left[ (1-\pi _{ij}) \cdot (Z_{ij} - \mu _{ij}) \right] (\boldsymbol{x}_{i} + \boldsymbol{x}_{j}), \\ \nabla _{\boldsymbol{\beta }}^{2} \mathop {\ln \mathcal {L}}\nolimits (\boldsymbol{\beta }\mid \boldsymbol{Z})&= -2 \sum _{\{i,j\}} \left[ (Z_{ij}+r) \pi _{ij} (1-\pi _{ij}) (\boldsymbol{x}_{i} + \boldsymbol{x}_{j}) \boldsymbol{x}_{j}^{\top } \right] , \\ \mathcal {I}(\boldsymbol{\beta })&= 2 \sum _{\{i,j\}} r \pi _{ij} \boldsymbol{x}_{i}\boldsymbol{x}^{\top }_{j} + r \pi _{ij}\boldsymbol{x}_{j}\boldsymbol{x}^{\top }_{j} = 2r \boldsymbol{X}\left[ \boldsymbol{\pi }+ \textrm{Diag}(\boldsymbol{\pi }\boldsymbol{1})\right] \boldsymbol{X}^{\top }. \end{aligned} \end{aligned}$$

Computing $\mathcal {I}(\boldsymbol{\beta })$ from $\nabla _{\boldsymbol{\beta }}^{2}\mathcal {L}(\boldsymbol{\beta }\mid \boldsymbol{Z})$ invokes the definition $\mu _{ij} = \mathrm{E\,\!}[Z_{ij}]$ and the convention $\mu _{ii} = Z_{ii} = 0$ adopted earlier. For fixed $\boldsymbol{\beta }$, one can update the *r* or $\alpha$ parameters via (7) to guarantee an increase in the log-likelihood.

### A few remarks on implementation, uncertainty, and inference

Implementing a practical algorithm to fit Poisson multigraphs is straightforward because it amounts to implementing (4) or (9) in conjunction with (11). Storing the expectations $\mu _{ij}$ simplifies matters by allowing one easily evaluate the current estimate’s log-likelihood. Fitting a negative binomial multigraph, however, is slightly more challenging as it involves cycling between updates to parameters of interest, such as (6) or (9), and updates to a dispersion parameter. Unfortunately, Fisher scoring (9) is liable to decrease the log-likelihood after updating coefficient estimates. In practice, we found it useful to implement aggressive backtracking by modifying the step size via the rule $t_{n} \mapsto 2^{-k} t_{n}$ for $k = 0,1,\ldots ,32$. In either case, we assess convergence of our algorithms via the relative change condition

$$\begin{aligned} \mathop {\ln \mathcal {L}}\nolimits (\boldsymbol{\theta }_{\text {new}} \mid \boldsymbol{Z}) - \mathop {\ln \mathcal {L}}\nolimits (\boldsymbol{\theta }_{\text {old}} \mid \boldsymbol{Z}) \le \epsilon [1 + \mathop {\ln \mathcal {L}}\nolimits (\boldsymbol{\theta }_{\text {old}})], \end{aligned}$$

for some positive $\epsilon$.

A more subtle implementation issue is initialization. The method of moments estimator of Ranola et al. can be used to seed propensities in the Poisson model [8]; for completeness the estimator is

$$\begin{aligned} p_{k} = \sqrt{ \frac{\sum _{\{i,j\}}~Z_{ij}}{n(n-1)}. } \end{aligned}$$

If the Poisson model involves node-specific attributes, we start from the method of moments estimator and apply a few iterations of the algorithm map in Eq. (4). Given the coarse propensity estimates, we then set $y_{i} = \log p_{i}$ and solve the linear system $\boldsymbol{y}= \boldsymbol{X}\boldsymbol{\beta }_{0}$ to seed the initial coefficient estimates, $\boldsymbol{\beta }_{0}$. We apply a similar strategy for the negative binomial models. Specifically, the propensity-based Poisson model estimates are used as a starting point for propensities in the propensity-based negative binomial model. The propensity-based negative binomial model in turn is used to seed its covariate-based counterpart. Because the scale parameter *r* is multimodal, our initial guess combines a method of moments estimator

$$\begin{aligned} r_{0} = {\left\{ \begin{array}{ll} \frac{\bar{z}^{2}}{s^{2} - \bar{z}}, & \text {if}~s^{2}-\bar{z}\\ 1, & \text {otherwise} \end{array}\right. } \end{aligned}$$

with a coarse logarithmic grid search around $r_{0}$. Here $\bar{z}$ and $s^{2}$ are the sample average and unbiased sample variance, respectively.

Our algorithms iteratively estimate propensity or predictor coefficients in parametric multigraph models. Thus, our estimating equations, which are of the form $\hat{\boldsymbol{\theta }} = \textrm{argmax}_{\boldsymbol{\theta }} \mathop {\ln \mathcal {L}}\nolimits (\boldsymbol{\theta }\mid \boldsymbol{Z})$, belong to the class of *M*-estimators and, more generally, extremum estimators. We use the expected information matrix for propensity parameters, $\mathcal {I}(\hat{\boldsymbol{p}})$, to coarsely approximate standard errors (SE) as $\text {SE}(\hat{p}_{i}) \approx \sqrt{[\mathcal {I}^{-1}(\hat{\boldsymbol{p}})]_{ii}}$.

The issue of increasing unknown parameters disappears in the covariate-based models. In this setting, the smoothness of our multigraph log-likelihoods guarantees asymptotic consistency as the number of nodes *n* increases, provided $\max _{\boldsymbol{\beta }}~\mathop {\ln \mathcal {L}}\nolimits (\boldsymbol{\beta }\mid \boldsymbol{Z})$ possess a unique solution or its solutions are well-separated. In this case, the expected information $\mathcal {I}(\hat{\boldsymbol{\beta }})$ provides reasonable standard errors.

Lastly, we address the issue of inferring significant associations between node pairs. Our approach relies on a mean-matching variance-stabilizing transformation *h*(*x*) for the statistics

13 $$\begin{aligned} S_{ij} = h(Z_{ij}) - \left[ h(\hat{\mu }_{ij}) + \frac{1}{2}h^{\prime \prime }(\hat{\mu }_{ij}) \textrm{Var}(Z_{ij})\right] , \end{aligned}$$

from which we compute *p*-values based on an observed value $Z_{ij} = z$, $\Pr (S_{ij} \ge |z|)$, for each pair of nodes. We use the choices $h(t) = 2 \sqrt{t + \frac{1}{4}}$ and $h(t) = 2 \sqrt{t + \frac{1}{4}\frac{\mu + r}{r}}$ for the Poisson and negative binomial models, respectively [15]. These choices imply $S_{ij} \sim \mathcal {N}(0,1)$. Given a matrix $\boldsymbol{P}$ of *p*-values, we then compute *mutual ranks*

14 $$\begin{aligned} M_{ij} = \left[ M_{ij}^{\text {row}}(\boldsymbol{P}) \cdot M_{ij}^{\text {col}}(\boldsymbol{P})\right] ^{1/2}, \end{aligned}$$

which are geometric averages of ranks for each edge pair based on rows, $M_{ij}^{\text {row}}(\boldsymbol{P})$, and columns, $M_{ij}^{\text {col}}(\boldsymbol{P})$. Mutual ranks capture associations between nodes in an interpretable and symmetric manner. The mutual rank approach has been proposed in bioinformatics literature for gene expression analysis [16], albeit based on correlation matrices. In the upcoming section, Figs. 2 through 4 report *p*-values on the mutual rank scale to emphasize relative strengths of associations between nodes as opposed to making a determination about whether the associations exist.

### Summary

We present a framework for modeling co-occurrences between entities using different edge distributions for a random multigraph. Our models have two main variations:

1. A *propensity*-based model in which observed edge counts $Z_{ij}$ between nodes *i* and *j* are modeled via $\mathrm{E\,\!}[Z_{ij}] = p_{i} p_{j}$. Here the $p_{j}$ are non-negative parameters to be estimated which quantify the likelihood that node *j* forms edges.
2. A *covariate*-based models the conditional expectation $\mathrm{E\,\!}[Z_{ij} \mid \boldsymbol{x}_{i}, \boldsymbol{x}_{j}] = p_{i}p_{j}$ in which one is given node-specific attributes $\boldsymbol{x}_{i}$ and $\boldsymbol{x}_{j}$ for nodes *i* and *j*, respectively. Covariate effects on node propensity $p_{j}$ are captured via regression, $\mathrm{E\,\!}[p_{j} \mid \boldsymbol{x}_{j}] = e^{\boldsymbol{x}_{j}^{\top } \boldsymbol{\beta }}$.

The two variants are presented for specific distributions on the $Z_{ij}$, namely Poisson and negative binomial, and can be extended to other distributions with finite means and variances. In either case, we investigate potential deviations from the propensity-based network dynamics by examining the statistics $S_{ij}$ defined in Eq. (13) and computing their associated *p*-values. Since these statistics grow as the number of nodes grows, we focus on relative importance of node associations by passing from *p*-values to the mutual rank scale as defined in Eq. (14). This has the benefit of emphasizing potentially interesting node associations while de-emphasizing the arbitrarily small *p*-values that may emerge in large networks.

## Results

### Overview

Our models consider graphs in which nodes *i* and *j* have multiple edges between them, each representing a repeated interaction or co-occurrence. The number of multi-edges between *i* and *j*, denoted $Z_{ij}$, is of primary interest. As an example, we consider co-expression patterns of genes in a particular organism. We take each gene as a node in a graph and draw an edge between them each time they are observed to co-express within a particular sample. Regardless of the application, it is crucial that the data $Z_{ij}$ represent counts.

Given edge data $Z_{ij}$ across *m* nodes, we form the $m \times m$ data matrix $\boldsymbol{Z}$. As a convention, we set the diagonal terms, $Z_{ii}$, equal to 0 so that the *j*-th column sum $\sum _{i=1}^{m} Z_{ij} = \sum _{i \ne j}^{m} Z_{ij}$; similarly for the row sums.

### Simulation studies

Here we describe several simulation studies in which we simulate multigraph data from a specific generative model and fit various multigraph models. We report summary statistics, based on 100 replicates, of various metrics by which to evaluate our algorithms and the fitted models: (i) average time spent running an algorithm in microseconds (ms), (ii) average number of iterations to achieve convergence as determined by a relative tolerance $\epsilon = 10^{-6}$, (iii) the average final negative log-likelihood value, (iv) the average of the mean squared error (MSE) of fitted propensities $\hat{\boldsymbol{p}}$ with respect to the ground truth $\boldsymbol{p}$, (v) the average MSE of expected counts $\hat{\boldsymbol{\mu }}$ given the fitted propensities, (vi) the average MSE of fitted coefficient $\hat{\boldsymbol{\beta }}$, and (vii) the median fitted scale parameter $\hat{r}$. Crucially, each simulation scenario fixes the propensities $\boldsymbol{p}$ and/or coefficients $\boldsymbol{\beta }$ and varies the observed data $Z_{ij}$ across replicates so as to maintain interpretability of the reported summary statistics. The purpose of these simulation studies is to demonstrate that our algorithms can recover reasonable estimates of model parameters, given multigraph data $\boldsymbol{Z}$, when matched to the correct model.

To elaborate on the simulation setup, in each scenario we sample *m* propensities as $p_{j} \sim \textrm{Uniform}(2,4)$ so as to construct a multigraph on *m* nodes. The sampled propensities are then used to compute expected counts $\mu _{ij} = p_{i} p_{j}$ from which we then simulate edge count data $Z_{ij}$ from a particular distribution. To study the covariate-based models, we generate a $p \times m$ covariate matrix $\boldsymbol{X}$ by sampling $x_{ij} \sim \mathcal {N}(0,1)$ and then standardize each row $\boldsymbol{x}_{i}$ to have mean 0 and variance 1. We sample predictor coefficients $\boldsymbol{\beta }$ by solving a least squares system, $\textrm{argmin}_{\boldsymbol{\beta }}~\Vert \boldsymbol{y}- \boldsymbol{X}\boldsymbol{\beta }\Vert ^{2}$, with response $y_{i} = \log _{10} m \cdot \log q_{i}$ and $q_{i} \sim \textrm{Uniform}(2,4)$ for $i=1,2,\ldots ,m$. This heuristic simulates coefficients $\boldsymbol{\beta }$ such that each propensity $p_{j}$ lies within or close to the interval [2, 4]. Each node’s propensity is then set by the rule $p_{i} = e^{\boldsymbol{x}_{i}^{\top }\boldsymbol{\beta }}$. The expected counts $\mu _{ij}$ and data $Z_{ij}$ are simulated as previously described.

Before proceeding, we note that Tables 1 through 4 report negative log-likelihoods, $-\mathop {\ln \mathcal {L}}\nolimits$, scaled by the total number of possible node pairs, $[m(m-1)]^{-1}$. The columns labeled *k* count the number of estimated parameters in fitting a multigraph model. The Akaike Information Criterion (AIC) is reported for each model fit, which takes the form of $-2[m(m-1)]^{-1}\mathop {\ln \mathcal {L}}\nolimits + 2k$. Finally, we denote the MSE of an estimate $\hat{\boldsymbol{\theta }}$ with respect to the ground truth $\boldsymbol{\theta }$ by $\textrm{MSE}(\hat{\boldsymbol{\theta }}) \equiv d^{-1} \sum _{i=1}^{d} (\hat{\theta }_{i} - \theta _{i})^{2}$.

### Simulating under the Poisson assumption

**Table 1:** Model fit summaries for 100 replicates of fitting simulated Poisson data with *m* nodes

	Poisson					Negative Binomial					
*m*	*k*	$-\mathop {\ln \mathcal {L}}\nolimits$	$\text {AIC}$	$\text {MSE}(\hat{\boldsymbol{p}})$	$\text {MSE}(\hat{\boldsymbol{\mu }})$	*k*	$-\mathop {\ln \mathcal {L}}\nolimits$	$\text {AIC}$	$\text {MSE}(\hat{\boldsymbol{p}})$	$\text {MSE}(\hat{\boldsymbol{\mu }})$	$\hat{r}$
**8**	**8**	**2.37 (0.11)**	**20.73**	**0.144 (0.079)**	**2.15 (1.2)**	9	2.37 (0.11)	22.73	0.144 (0.079)	2.15 (1.2)	21223
**16**	**16**	**2.41 (0.057)**	**36.83**	**0.062 (0.021)**	**1.01 (0.33)**	17	2.41 (0.056)	38.83	0.0622 (0.021)	1.01 (0.33)	23841
**32**	**32**	**2.44 (0.027)**	**68.88**	**0.0326 (0.0088)**	**0.552 (0.15)**	33	2.44 (0.027)	70.88	0.0326 (0.0088)	0.553 (0.15)	17958
**64**	**64**	**2.45 (0.014)**	**132.9**	**0.0163 (0.003)**	**0.286 (0.052)**	65	2.45 (0.014)	134.9	0.0163 (0.003)	0.287 (0.052)	14756
**128**	**128**	**2.46 (0.0071)**	**260.9**	**0.00769 (0.001)**	**0.136 (0.018)**	129	2.46 (0.0071)	262.9	0.0077 (0.001)	0.136 (0.018)	13676
**256**	**256**	**2.47 (0.0039)**	**516.9**	**0.00393 (0.00035)**	**0.0714 (0.0062)**	257	2.47 (0.0039)	518.9	0.00394 (0.00035)	0.0715 (0.0062)	13568
**512**	**512**	**2.48 (0.0022)**	**1029**	**0.00192 (0.00012)**	**0.0353 (0.0021)**	513	2.48 (0.0022)	1031	0.00193 (0.00012)	0.0354 (0.0021)	13228

The generative model is in bold. All values are averages with standard deviations in parentheses, except for$\hat{r}$which is reported as a median. Here *k* indicates the number of parameters factoring into a model’s AIC

Table 1 compares representative model fits for Poisson distributed data under the Poisson and negative binomial algorithms. There is no obvious winner between the two algorithms as the fitted Poisson models barely outperform the fitted negative binomial models in terms of negative log-likelihood, MSE of propensities, and MSE of expected edge counts. In defense of the Poisson algorithm, the fitted value for the scale parameter, $\hat{r}$, tends to be large which puts the fitted negative binomial counts in the Poisson regime. In addition, the Poisson algorithm is fast in comparison.

**Table 2:** Model fit summaries for 100 replicates of fitting to simulated Poisson data with $m=32$ nodes and *p* covariates

	Poisson (Propensity)						Negative Binomial (Propensity)						
*p*	*k*	$-\mathop {\ln \mathcal {L}}\nolimits$	$\text {AIC}$	$\text {MSE}(\hat{\boldsymbol{p}})$	$\text {MSE}(\hat{\boldsymbol{\mu }})$	$\text {MSE}(\hat{\boldsymbol{\beta }})$	*k*	$-\mathop {\ln \mathcal {L}}\nolimits$	$\text {AIC}$	$\text {MSE}(\hat{\boldsymbol{p}})$	$\text {MSE}(\hat{\boldsymbol{\mu }})$	$\text {MSE}(\hat{\boldsymbol{\beta }})$	$\hat{r}$
1	32	2.44 (0.029)	68.9	0.0327 (0.0077)	0.553 (0.13)		33	2.44 (0.029)	70.9	0.0327 (0.0077)	0.553 (0.13)		18114
2	32	2.44 (0.03)	68.9	0.0330 (0.0075)	0.557 (0.13)		33	2.44 (0.029)	70.9	0.0330 (0.0075)	0.557 (0.13)		17733
4	32	2.44 (0.025)	68.9	0.0321 (0.0093)	0.540 (0.16)		33	2.44 (0.025)	70.9	0.0321 (0.0093)	0.540 (0.16)		18060
8	32	2.44 (0.029)	68.9	0.0330 (0.0084)	0.558 (0.14)		33	2.44 (0.028)	70.9	0.0330 (0.0084)	0.559 (0.14)		17407
16	32	2.44 (0.031)	68.9	0.0314 (0.0069)	0.533 (0.12)		33	2.44 (0.03)	70.9	0.0315 (0.0069)	0.533 (0.12)		18050

	Poisson (Covariate)						Negative Binomial (Covariate)						
*p*	*k*	$-\mathop {\ln \mathcal {L}}\nolimits$	$\text {AIC}$	$\text {MSE}(\hat{\boldsymbol{p}})$	$\text {MSE}(\hat{\boldsymbol{\mu }})$	$\text {MSE}(\hat{\boldsymbol{\beta }})$	*k*	$-\mathop {\ln \mathcal {L}}\nolimits$	$\text {AIC}$	$\text {MSE}(\hat{\boldsymbol{p}})$	$\text {MSE}(\hat{\boldsymbol{\mu }})$	$\text {MSE}(\hat{\boldsymbol{\beta }})$	$\hat{r}$
**1**	**33**	**2.44 (0.029)**	**70.9**	**0.0327 (0.0077)**	**0.553 (0.13)**	**0.00451 (0.0014)**	34	2.44 (0.029)	72.9	0.0327 (0.0077)	0.553 (0.13)	0.00459 (0.0014)	18114
**2**	**34**	**2.44 (0.03)**	**72.9**	**0.0330 (0.0075)**	**0.557 (0.13)**	**0.00542 (0.0022)**	35	2.44 (0.029)	74.9	0.0330 (0.0075)	0.557 (0.13)	0.00557 (0.0023)	17733
**4**	**36**	**2.44 (0.025)**	**76.9**	**0.0321 (0.0093)**	**0.540 (0.16)**	**0.00653 (0.0025)**	37	2.44 (0.025)	78.9	0.0321 (0.0093)	0.540 (0.16)	0.00682 (0.0027)	18060
**8**	**40**	**2.44 (0.029)**	**84.9**	**0.0330 (0.0084)**	**0.559 (0.14)**	**0.00867 (0.0027)**	41	2.44 (0.028)	86.9	0.0330 (0.0084)	0.559 (0.14)	0.00916 (0.0029)	17407
**16**	**48**	**2.44 (0.031)**	**101**	**0.0315 (0.0069)**	**0.533 (0.12)**	**0.0115 (0.0029)**	49	2.44 (0.03)	103	0.0315 (0.0069)	0.533 (0.12)	0.0123 (0.0031)	18050

The generative model is in bold. All values are averages with standard deviations in parentheses, except for$\hat{r}$which is reported as a median. Here *k* indicates the number of parameters factoring into a model’s AIC

Table 2 reports model fits for both propensity and covariate versions of the Poisson and negative binomial models. Both covariate models recover good estimates of propensity values on the basis of mean squared error compared to the propensity models. In addition, both the Poisson and negative binomial models with covariates achieve reasonable estimates of model coefficients. The Poisson covariate model is appreciably fast to converge, requiring as few as 2 iterations. The only concern for the covariate models is the larger negative log-likelihood values compared to the propensity-based versions. As before, we note that the negative binomial with covariates operates in the Poisson regime with large $\hat{r}$ values. Overall, our results in Table 2 suggest that including node-specific attributes both improves propensity estimates and offers insight into potential mechanisms driving associations between node entities.

### Simulating under the negative binomial assumption

**Table 3:** Model fit summaries after fitting to simulated negative binomial data with *m* nodes

		Poisson					Negative Binomial					
*m*	*r*	*k*	$-\mathop {\ln \mathcal {L}}\nolimits$	$\text {AIC}$	$\text {MSE}(\hat{\boldsymbol{p}})$	$\text {MSE}(\hat{\boldsymbol{\mu }})$	*k*	$-\mathop {\ln \mathcal {L}}\nolimits$	$\text {AIC}$	$\text {MSE}(\hat{\boldsymbol{p}})$	$\text {MSE}(\hat{\boldsymbol{\mu }})$	$\hat{r}$
8	0.5	8	5.94 (1.7)	27.9	3.44 (2.6)	57.2 (49)	**9**	**2.99 (0.33)**	**24.0**	**4.21 (3)**	**70.6 (56)**	**0.79**
8	1.0	8	4.49 (0.84)	25.0	1.57 (0.92)	24.6 (17)	**9**	**3.05 (0.22)**	**24.1**	**1.72 (1.1)**	**27.1 (22)**	**1.6**
8	10.0	8	2.68 (0.21)	21.4	0.284 (0.15)	4.33 (2.4)	**9**	**2.63 (0.15)**	**23.3**	**0.285 (0.15)**	**4.34 (2.4)**	**30**
16	0.5	16	7.19 (0.86)	46.4	1.39 (0.44)	24.8 (9.1)	**17**	**3.06 (0.14)**	**40.1**	**1.55 (0.6)**	**27.8 (11)**	**0.6**
16	1.0	16	5.11 (0.53)	42.2	0.678 (0.25)	11.3 (4.5)	**17**	**3.12 (0.1)**	**40.2**	**0.701 (0.24)**	**11.6 (4.4)**	**1.2**
16	10.0	16	2.78 (0.099)	37.6	0.133 (0.051)	2.2 (0.85)	**17**	**2.71 (0.061)**	**39.4**	**0.133 (0.052)**	**2.2 (0.87)**	**15**
32	0.5	32	7.57 (0.48)	79.1	0.638 (0.19)	11.1 (3.5)	**33**	**3.05 (0.067)**	**72.1**	**0.642 (0.2)**	**11.2 (3.7)**	**0.55**
32	1.0	32	5.42 (0.26)	74.8	0.331 (0.1)	5.64 (1.8)	**33**	**3.14 (0.046)**	**72.3**	**0.325 (0.099)**	**5.54 (1.7)**	**1.1**
32	10.0	32	2.81 (0.059)	69.6	0.0601 (0.015)	1.02 (0.26)	**33**	**2.71 (0.033)**	**71.4**	**0.06 (0.015)**	**1.01 (0.26)**	**13**

The generative model is in bold. All values are averages with standard deviations in parentheses, except for$\hat{r}$which is reported as a median. Here *k* indicates the number of parameters factoring into a model’s AIC

Following our simulation study in Sect. "Simulating under the Poisson assumption", we change the underlying distribution of our generative model to the negative binomial. To investigate robustness to overdispersion, we simulate negative binomial edge counts with $r \in \{0.5, 1, 10\}$. Given that each propensity $p_{j} \in [2,4]$, the theoretical expected counts are bounded as $4 \le \mu _{ij} \le 16$. Hence, the cases with $r=0.5$ and $r=1$ correspond to moderate and weak dispersion, respectively.

**Table 4:** Model fit summaries after fitting to simulated negative binomial data with $m=32$ nodes and *p* covariates

		Poisson (Propensity)						Negative Binomial (Propensity)						
*p*	*r*	*k*	$-\mathop {\ln \mathcal {L}}\nolimits$	$\text {AIC}$	$\text {MSE}(\hat{\boldsymbol{p}})$	$\text {MSE}(\hat{\boldsymbol{\mu }})$	$\text {MSE}(\hat{\boldsymbol{\beta }})$	*k*	$-\mathop {\ln \mathcal {L}}\nolimits$	$\text {AIC}$	$\text {MSE}(\hat{\boldsymbol{p}})$	$\text {MSE}(\hat{\boldsymbol{\mu }})$	$\text {MSE}(\hat{\boldsymbol{\beta }})$	$\hat{r}$
1	0.5	32	7.57 (0.54)	79.1	0.645 (0.18)	11.2 (3.4)		33	3.04 (0.061)	72.1	0.690 (0.21)	12.1 (4.1)		0.54
1	1.0	32	5.42 (0.25)	74.8	0.329 (0.098)	5.59 (1.7)		33	3.14 (0.044)	72.3	0.335 (0.098)	5.71 (1.7)		1.1
1	10.0	32	2.81 (0.061)	69.6	0.0610 (0.017)	1.04 (0.30)		33	2.71 (0.035)	71.4	0.0609 (0.017)	1.03 (0.30)		12
4	0.5	32	7.59 (0.54)	79.2	0.619 (0.15)	10.9 (3.0)		33	3.06 (0.063)	72.1	0.623 (0.15)	11.0 (3.0)		0.55
4	1.0	32	5.48 (0.27)	75	0.324 (0.080)	5.55 (1.4)		33	3.15 (0.043)	72.3	0.324 (0.080)	5.55 (1.4)		1.1
4	10.0	32	2.80 (0.062)	69.6	0.0620 (0.015)	1.06 (0.25)		33	2.71 (0.036)	71.4	0.0612 (0.014)	1.04 (0.24)		12
16	0.5	32	7.57 (0.58)	79.1	0.627 (0.18)	11.0 (3.6)		33	3.05 (0.071)	72.1	0.650 (0.17)	11.5 (3.5)		0.54
16	1.0	32	5.43 (0.27)	74.9	0.339 (0.097)	5.74 (1.6)		33	3.14 (0.047)	72.3	0.337 (0.098)	5.71 (1.6)		1.1
16	10.0	32	2.80 (0.061)	69.6	0.0639 (0.016)	1.09 (0.27)		33	2.71 (0.037)	71.4	0.0635 (0.016)	1.08 (0.27)		12

		Poisson (Covariate)						Negative Binomial (Covariate)						
*p*	*r*	*k*	$-\mathop {\ln \mathcal {L}}\nolimits$	$\text {AIC}$	$\text {MSE}(\hat{\boldsymbol{p}})$	$\text {MSE}(\hat{\boldsymbol{\mu }})$	$\text {MSE}(\hat{\boldsymbol{\beta }})$	*k*	$-\mathop {\ln \mathcal {L}}\nolimits$	$\text {AIC}$	$\text {MSE}(\hat{\boldsymbol{p}})$	$\text {MSE}(\hat{\boldsymbol{\mu }})$	$\text {MSE}(\hat{\boldsymbol{\beta }})$	$\hat{r}$
1	0.5	33	7.57 (0.54)	81.1	0.645 (0.18)	11.2 (3.4)	0.0756 (0.021)	**34**	**3.04 (0.061)**	**74.1**	**0.694 (0.21)**	**12.2 (4.2)**	**0.0796 (0.023)**	**0.54**
1	1.0	33	5.42 (0.25)	76.8	0.329 (0.098)	5.59 (1.7)	0.0382 (0.011)	**34**	**3.14 (0.044)**	**74.3**	**0.336 (0.098)**	**5.73 (1.7)**	**0.0390 (0.011)**	**1.1**
1	10.0	33	2.81 (0.061)	71.6	0.0610 (0.017)	1.04 (0.30)	0.00772 (0.0023)	**34**	**2.71 (0.035)**	**73.4**	**0.0609 (0.017)**	**1.03 (0.30)**	**0.00788 (0.0022)**	**12**
4	0.5	36	7.59 (0.54)	87.2	0.619 (0.15)	10.9 (3.0)	0.0662 (0.016)	**37**	**3.06 (0.063)**	**80.1**	**0.627 (0.15)**	**11.1 (3.0)**	**0.0669 (0.016)**	**0.55**
4	1.0	36	5.48 (0.27)	83	0.324 (0.080)	5.55 (1.4)	0.0363 (0.0090)	**37**	**3.15 (0.043)**	**80.3**	**0.325 (0.080)**	**5.57 (1.4)**	**0.0363 (0.0088)**	**1.1**
4	10.0	36	2.80 (0.062)	77.6	0.0620 (0.015)	1.06 (0.25)	0.00928 (0.0029)	**37**	**2.71 (0.036)**	**79.4**	**0.0612 (0.014)**	**1.04 (0.24)**	**0.00955 (0.0031)**	**12**
16	0.5	48	7.57 (0.58)	111	0.627 (0.18)	11.0 (3.6)	0.0568 (0.013)	**49**	**3.05 (0.071)**	**104**	**0.654 (0.17)**	**11.6 (3.5)**	**0.0571 (0.012)**	**0.54**
16	1.0	48	5.43 (0.27)	107	0.339 (0.097)	5.74 (1.6)	0.0352 (0.0091)	**49**	**3.14 (0.047)**	**104**	**0.338 (0.099)**	**5.73 (1.6)**	**0.0354 (0.0090)**	**1.1**
16	10.0	48	2.80 (0.061)	102	0.0640 (0.016)	1.09 (0.27)	0.0138 (0.0034)	**49**	**2.71 (0.037)**	**103**	**0.0635 (0.016)**	**1.08 (0.27)**	**0.0147 (0.0036)**	**12**

The generative model is in bold. All values are averages with standard deviations in parentheses, except for$\hat{r}$which is reported as a median. Here *k* indicates the number of parameters factoring into a model’s AIC

**Fig. 1:**
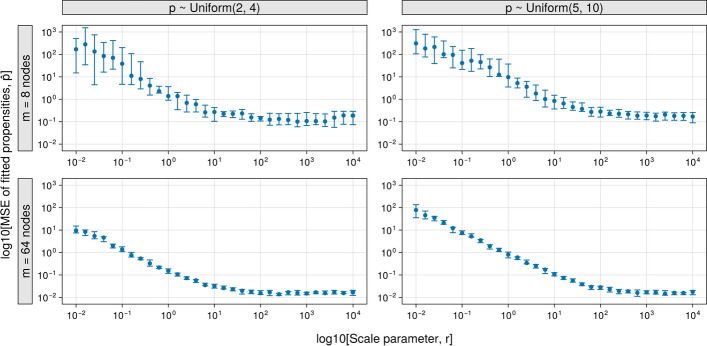
MSE of a propensities, fitted to edge count data under the negative binomial distribution, as a function of the scale parameter *r*. Top and bottom rows correspond to multigraphs with $m=8$ and $m=64$ nodes, respectively. Left and right columns correspond to propensities sampled as $p_{j} \sim \textrm{Uniform}(2,4)$ and $p_{j} \sim \textrm{Uniform}(5,10)$, respectively

Tables 3 and 4 report model fit summaries for the propensity and covariate versions of our multigraph models, respectively. In Table 3 it is apparent that the fitted negative binomial models outperform their Poisson counterparts on the basis of minimizing the negative log-likelihoods. This trend holds true as the number of nodes *m* increases and the scale parameter *r* shifts from the overdispersed regime (small *r*) to the Poisson regime (large *r*). Unfortunately, the large MSE$(\hat{\boldsymbol{p}})$ values indicate that fitted propensities are poor estimates of the ground truth. Inspection of the simulated data revealed that counts $Z_{ij}$ tend to be zero-inflated particularly in the overdispersed regime particularly when $r=0.5$. This effect is mitigated as the number of nodes increases, as seen by the decreasing MSE values. Decreasing the amount of dispersion by increasing *r* also reduces zero-inflation as evidenced by the MSE values where $r=10$ in Table 3. We validate the stated trends by simulating negative binomial multigraphs across a wider range of *r* values as reported in Fig. 1. Our results in Table 4 also indicate that accounting for node-specific attributes improve fitted propensity values in various overdispersion scenarios.

### Gene co-expression in *Saccharomyces cerevisiae*

**Fig. 2:**
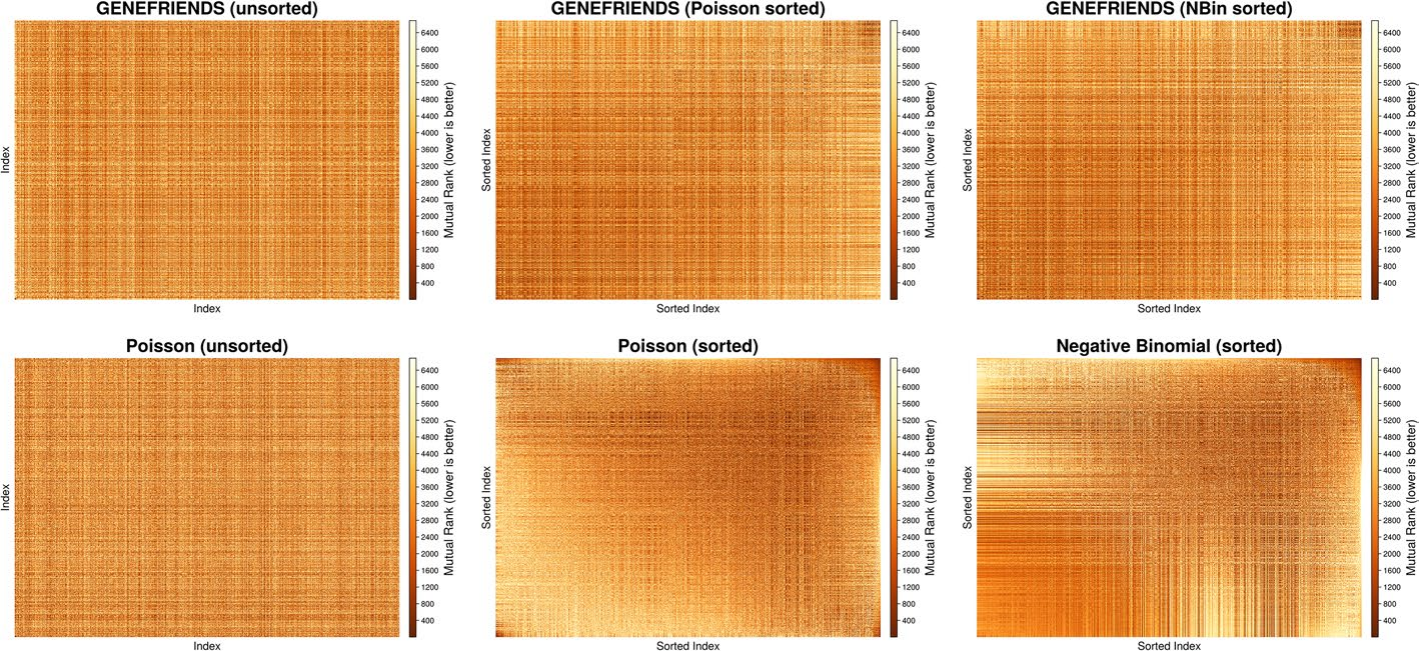
Qualitative comparison of correlation networks (top row) and propensity-based multigraphs (bottom row). Comparisons are to results obtained from the GeneFriends database and are reported on a mutual rank scale, with lower values (darker colors) indicating greater relative importance of an edge to the network. (Left) Genes appear in the original order given by the data along the *x* and *y* axis. Genes are sorted in descending order based on estimated propensities from the Poisson model (Middle) and negative binomial (Right)

Next we consider yeast RNA sequencing data archived in the ARCH$^4$ Zoo database [12]. This dataset contains expression profiles for 6689 specimens across 6690 genes. Following quantile normalization of $\log _{2}$ pseudocounts, each profile $\boldsymbol{x}\in \mathbb {R}^{6690}$ is converted into a binary vector $\boldsymbol{b}\in \{0,1\}^{6690}$ indicating whether gene *j* is expressed according to a threshold. We then count the number of samples expressing each gene to obtain a $6690 \times 6690$ count matrix $\boldsymbol{Z}$ indicating co-expression of two genes whenever $Z_{ij} > 0$.

Given $\boldsymbol{Z}$, we then fit Poisson and negative binomial models to the co-expression data. For comparison, we use the mutual rank matrices reported by the GeneFriends database [17]. The GeneFriends results are obtained by computing weighted Pearson correlations [18] followed by transforming the result to the mutual rank scale. Figure 2 visually reports the relative importance of edges in the gene network using the mutual rank scale. In the 2nd and 3rd columns of Fig. 2, we reorder nodes in descending order using the propensities obtained from the corresponding multigraph model. This maneuver groups nodes with high and low propensities at the extremes of the axes, revealing additional structure not available using only the mutual ranks. For example, the top right corner of Fig. 2 has a module of low propensity genes revealed by sorting using the negative binomial’s fitted propensities. The visual patterns suggest that multigraph models offer a different perspective for analyzing co-expression patterns.

**Table 5:** Summary of the largest 3 connected components extracted using mutual rank matrices for the GeneFriends, Poisson, and negative binomial models

	GeneFriends			Poisson			Negative Binomial		
total	495			105			114		
component	1	2	3	1	2	3	1	2	3
size	25	16	14	120	93	22	112	49	46
min	48.1	48.2	6.16	3.54	1.34	6.85	3.54	2.04	6.85
mean	53.8	53.8	16.6	22.0	8.09	20.4	21.8	8.95	19.7
std	2.19	2.84	6.79	9.01	7.82	8.26	10.2	12.7	5.71
max	56.6	56.2	27.6	42.2	44.6	41.5	42.2	49.3	30.3
min	47.0	47.5	6.41	3.71	1.4	6.88	3.71	2.26	6.88
mean	52.9	52.9	17.6	22.1	8.48	20.9	22.0	9.23	20.2
std	2.32	2.89	6.7	8.63	7.71	8.34	9.71	12.3	5.81
max	56.0	55.4	28.2	41.5	43.9	41.8	41.5	48.3	31.2

Row Group 1: Total number, component label, and component size.Row Group 2: Descriptive statistics for Poisson propensities associated with each gene in the indicated component.Row Group 3: Descriptive statistics for negative binomial propensities associated with each gene in the indicated component.

The patterns in Fig. 2 suggest that one might obtain clusters of genes by constructing a sparse graph from the mutual rank matrices $\boldsymbol{M}= (M_{ij})$. This can be achieved by applying a threshold to the mutual ranks and assigning a value of 1 to any entries that fall below the threshold value, and 0 otherwise. Our empirical investigation suggests that $M_{ij} \le 3$ maximizes the number of non-trivial connected components (n Information). Table 5 records the total number of connected components and the sizes of the top 3 largest components obtained from the GeneFriends, Poisson, and negative binomial mutual ranks. We also report descriptive statistics for propensity values based on the nodes in each of the top 3 components. The multigraph models induce fewer connected components and greater propensity diversity compared to the correlation-based approach. Table 6 reports the results of a KEGG Pathway enrichment analysis for the top 3 components under each model, based on the DAVID functional annotation tool [19]. The multigraph model results are enriched for terms related to metabolism, whereas correlation analysis (GeneFriends) reveals components associated with protein processing. As such, mGEM analysis provides novel insights compared to correlation analysis.

**Table 6:** KEGG Pathway enrichment analysis of connected components derived from mutual rank matrices, using the DAVID functional annotation tool

GeneFriends	Size	DAVID IDs	Term	Count	*p*-value	Benjamini-Hochberg
Component 1	25	25	Protein processing in endoplasmic reticulum	3	0.069	1.0
Component 2	16	20	Ribosome	20	$2.1 \times 10^{-22}$	$4.2 \times 10^{-22}$
Component 3	14	5	Ribosome	4	$4.3 \times 10^{-4}$	$4.3 \times 10^{-4}$
Component 1	120	118	Carbon metabolism	8	$8.7 \times 10^{-4}$	0.043
			Metabolic pathways	21	$2.5 \times 10^{-3}$	0.062
			Methane metabolism	4	$5.6 \times 10^{-3}$	0.091
			Pyruvate metabolism	4	0.037	0.042
			Glycolysis/Gluconeogenesis	4	0.043	0.42
			Citrate cycle (TCA cycle)	3	0.072	0.58
Component 2	93	63	Meiosis (yeast)	4	0.022	0.36
Component 3	22	22	Oxidative phosphorylation	9	$2.9 \times 10^{-11}$	$1.1 \times 10^{-10}$
			Metabolic pathways	9	$2.4 \times 10^{-3}$	$4.8 \times 10^{-3}$
**Negative Binomial**	**Size**	**DAVID IDs**	**Term**	**Count**	**p-value**	**Benjamini-Hochberg**
Component 1	112	109	Carbon metabolism	8	$8.7 \times 10^{-4}$	0.043
			Peroxisome	5	$2.2 \times 10^{-3}$	0.055
			Methane metabolism	4	$5.6 \times 10^{-3}$	0.091
			Metabolic pathways	19	0.017	0.21
			Pyruvate metabolism	4	0.037	0.35
			Glycolysis/Gluconeogenesis	4	0.043	0.35
			Citrate cycle (TCA cycle)	3	0.072	0.44
			RNA polymerase	3	0.072	0.44
Component 2	49	23				
Component 3	46	23				

### *Caernorhabditis elegans* neural network

**Table 7:** Top 10 neuron connections in *C. elegans* under the Poisson and negative binomial models, scored and ranked by the negative base-10 logarithm of *p*-values (Score)

Poisson					Negative Binomial				
Neuron pair	Type	$Z_{ij}$	$\hat{\mu }_{ij}$	Score	Neuron pair	Type	$Z_{ij}$	$\hat{\mu }_{ij}$	Score
VB03, DD02	S	37	0.36	27	VB03, DD02	S	37	0.36	21
PDER, DVA	Sp	35	0.96	22	VB08, DD05	S	31	0.33	17
VB08, DD05	S	31	0.32	22	AVFR, AVFL	Sp	31	0.36	17
AVFR, AVFL	Sp	31	0.36	22	VB06, DD04	S	30	0.31	17
VB06, DD04	S	30	0.29	22	PDER, DVA	Sp	35	0.84	16
VB05, DD03	S	27	0.36	19	VB05, DD03	S	27	0.41	14
VD05, DB03	Rp	26	0.47	18	VD03, DA03	Rp	25	0.38	13
VD03, DA03	Rp	25	0.4	17	VD05, DB03	Rp	26	0.47	13
PDEL, DVA	Sp	27	0.76	17	VA06, DD03	S	24	0.31	13
VA06, DD03	S	24	0.4	16	VD03, DB02	Rp	23	0.37	12

We revisit the classic *C. elegans* neural network published by White et al. [13]. Briefly, the neural network consists of various synapses, which are necessarily directional connections, were identified in high-resolution electron microscope images. For our purposes, we combine directional connections by summing the number of synapses from neuron *i* to *j* and from *j* to *i*. Our analysis deviates slightly from the original multigraph analysis of Ranola et al. in that we ignore electric junctions and neuromuscular junctions identified by Chen et al. [20]. Table 7 illustrates our ranking procedure via our symmetrized *p*-values. The top 10 neuron pairs are similar across the Poisson and negative binomial models, and closely match those reported by Ranola et al. [8, see Table 1].

### Google Scholar co-authorship analysis

Recently, Kalhor et al. analyzed a subset of the Manually Added Co-authorship Network (MACN) of public Google Scholar users [14]. In a MACN author A is said to be a co-author of author B if A adds B to his or her list of research collaborators. This is in contrast to typical co-authorship networks which treat individuals as co-authors if their names appear together on a publication. Kalhor et al. contend that co-occurrence of author names may fail to capture direct research collaborations, especially in publications with hundreds of authors that are unlikely to all know each other [14]. Thus, the Google Scholar MACN represents an incomplete network of co-authorship, in terms of author name co-occurrence, but potentially captures stronger signals of scientific collaboration. The authors use the MACN to derive a Field of Interest Network (FIN) and an Affiliated Institution Network (AIN) to illustrate collaborations across disciplines and research institution, respectively. Both networks have edges weighted by the number of co-authorship relationships between nodes which can be interpreted as multi-edges. The FIN and AIN are the data of interest for our analysis. Critically, we note that the FIN and AIN we analyze are based 850,827 authors across 30 research institutions and 38 fields of interest. Each institution represents a single field as originally compiled by Kalhor et al. [14].

**Fig. 3:**
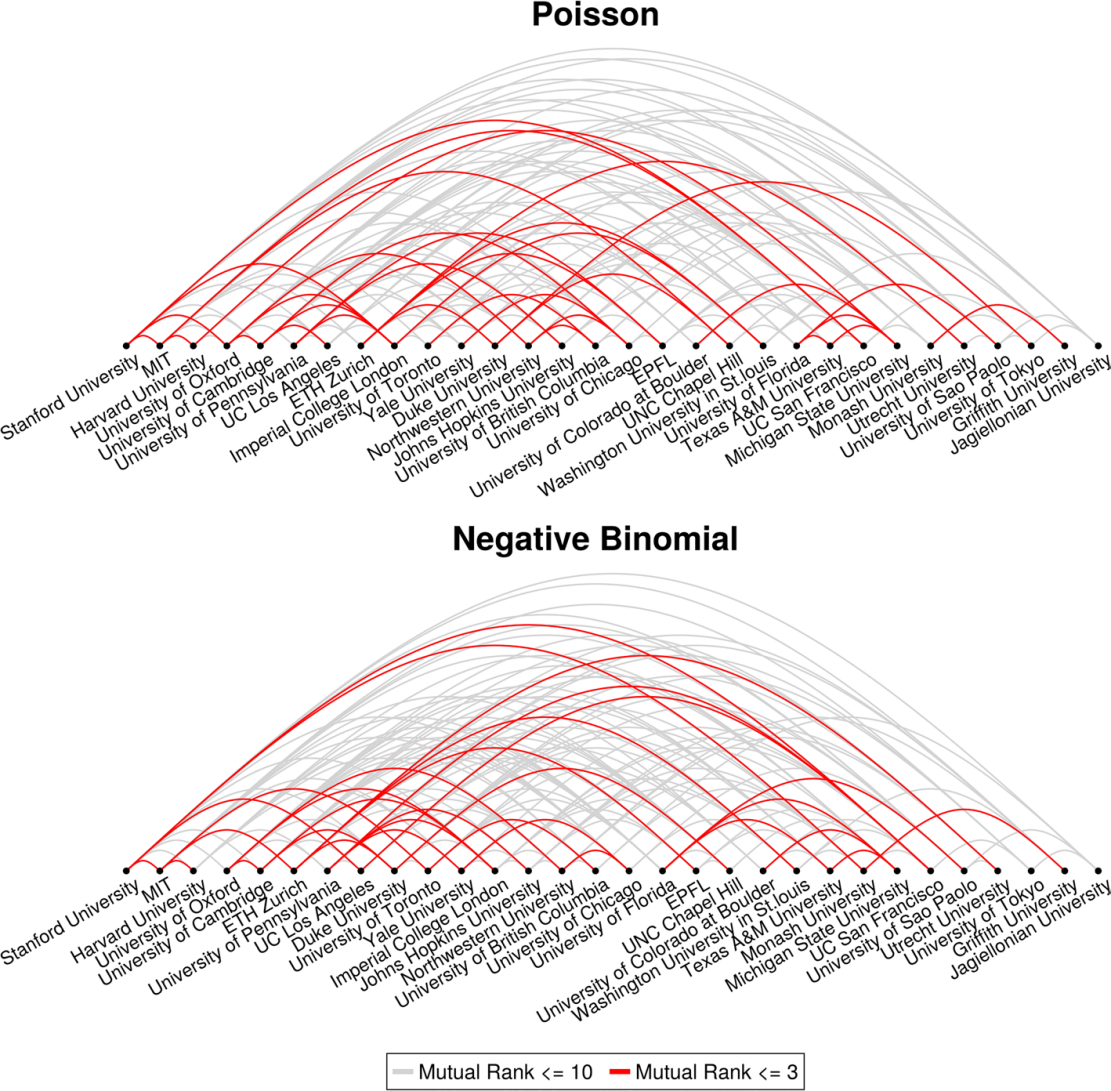
Strong associations between affiliated institutions (AIN) in the Google Scholar MACN, as implied by mutual ranking. Institutions are presented in descending propensity order from left (30.97) to right (0.4736)

In our analysis we fit propensity-based models to both the AIN and FIN and investigate both networks for highly correlated node interactions. Concretely, in the case of the AIN nodes represent different institutions and edges quantify the number of co-authorship relationships between them, as detected in the Google Scholar MACN. Thresholding the mutual rank at different levels induces an adjacency matrix which reveals information about scientific collaborations across research institutions. A similar interpretation holds for the case of the FIN, except nodes represent fields of interest rather than institutions. We also demonstrate how covariates are incorporated into our network analysis by fitting a covariate-based model to the FIN. In this setting, we rank fields of interest as in the propensity model setting while controlling for citation counts, *h*-index, and genders represented within each field.

Figure 3 visualizes the AIN graph implied by thresholding the mutual rank matrix at 10 and 3, the latter being sparser. Starting from the left hand side and moving towards the right, we report each AIN in order of decreasing propensities. The more restrictive graph (mutual rank $\le$ 3) suggests a few interesting groupings of research universities. For example, Duke, UNC Chapel Hill, and MIT are all linked and are located on the Eastern coast of the United States. Notably, their propensity values are not similar to each other. The reader might also note that the most connected universities are not necessarily those with large propensity values. The node for Imperial College London illustrates this point well.

**Fig. 4:**
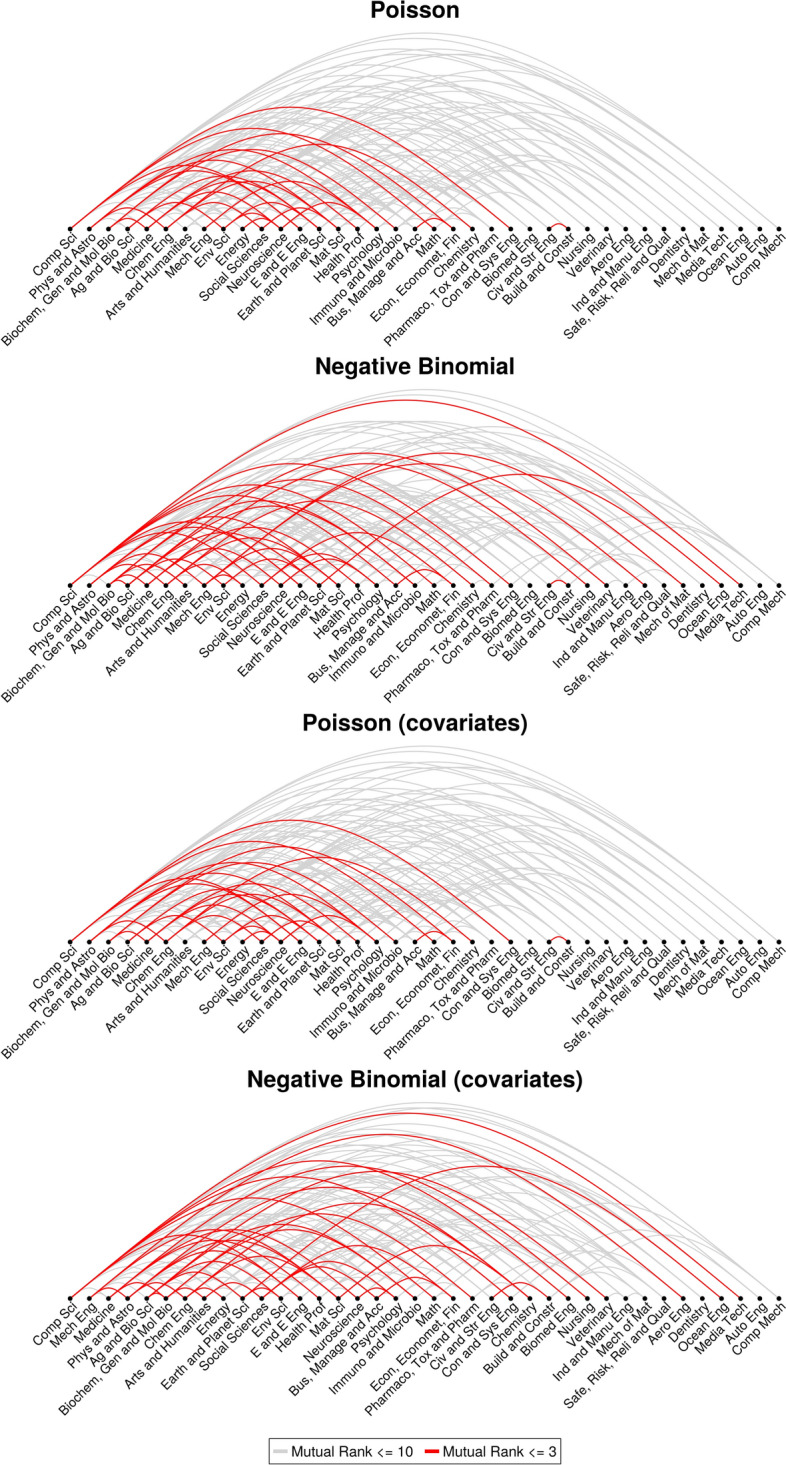
Strong associations between fields of interest (FIN) in the Google Scholar MACN, as implied by mutual ranking. Fields are presented in descending propensity order from left to right

Figure 4 suggests strong associations in the FIN multigraph using both the propensity and covariate models. “Computer Science” features prominently near the top but this is not too surprising as it is overrepresented in the dataset. Specifically, 8 out of 30 institutions in the AIN are taken as representatives for “Computer Science”. A few surprising changes occur in the rankings when adding covariates. For example, the fields “Neuroscience” and “Mechanical Engineering” move down and up in the rankings, respectively, in the negative binomial model after factoring information about citation counts, *h*-index, and gender. Many of the highlighted connections are expected; for example it is not unusual to see doctors (“Medicine”) publish alongside neuroscientists (“Neuroscience”). Table 8 summarizes the coefficients assigned to each variable considered in our models. Notably, signs are consistent between the Poisson and negative binomial models. Citation counts appear to have a negative effect on collaboration propensities. In summary, mGEM is capable of highlighting interesting patterns in co-occurrence data, thereby suggesting the possible shape of the underlying network. This capability is further enhanced by incorporating node-level attributes to modulate multigraph propensity values.

**Table 8:** Summary of coefficients in the covariate models for the FIN

Poisson		Negative Binomial	
	Coefficient (SE)		Coefficient (SE)
h_index_std	0.0653 (0.00091)	citations_mean	−0.64 (0.15)
citations_mean	−0.0626 (0.0017)	n_female	0.57 (0.12)
n_female	0.0362 (0.00077)	citations_std	0.55 (0.14)
n_male	−0.0197 (0.00073)	n_male	−0.45 (0.11)
h_index_mean	−0.0188 (0.001)	h_index_mean	0.13 (0.2)
citations_std	0.00846 (0.0012)	h_index_std	−0.03 (0.32)

## Discussion

The mGEM approach can be used as a null model for exploratory pattern detection and data analyses, as demonstrated by our examples of random multigraph models in two key paradigms (1) the propensity-based models which primarily concern the tendency of correlated nodes to interact, and (2) the covariate-based models in which specific attributes contribute to a node’s tendency to form edges. We also demonstrate the flexibility of the modelling assumptions in our random multigraph models, namely independence of nodes and conditional expectation conditions, (2) and (8), by highlighting how one can adapt the theory to the negative binomial distribution. Our simulation results in Tables 1 through 4 validate negative binomial multigraphs as a robust alternative to Poisson random multigraphs, as expected by the fact that the two distributions are nested. Indeed, any multiedge distribution with support on the non-negative integers is viable; for example, the geometric distribution is a special case of the mean-scale negative binomial with $r=1$. In view of the insights from Fig. 1, zero-inflated models are an important class of distributions for future consideration that are likely useful in analyzing large sparse networks. Extensions to other multiedge distributions should also account for clustering [10], as many real-world networks exhibit small-word or scale-free phenomena. Practitioners are doubtlessly interested in verifying the distributional assumptions regarding the related models and assessing the utility of modeling node-specific attributes. Tables 1 and 3 illustrate that (negative) log-likelihoods can be used to compare nested propensity-based models. We also show that AIC is useful in comparing between pure propensity and covariate models due to its penalty on model complexity.

We also demonstrated how to apply random multigraph analyses to real data such as the Google Scholar MACN. Combining our propensity-based models with regression provided insight into important institutions and fields of interest while simultaneously estimating effect sizes for various predictors that may drive collaborations. Our excursion into RNA-seq analysis also suggests that multigraph models may offer a useful perspective that complements existing correlation-based methods of pattern detection. Indeed, we remind readers that mGEM is primarily an exploratory tool and not a replacement for inferential tools, such as community detection or structure identification.

In future work, we hope to devise techniques to analyze networks that may contain node annotations but lack edge weights or a multigraph interpretation. We invite readers to join us in this endeavor.

## Data Availability

Software and documentation are available at the GitHub repository https://github.com/alanderos91/MultiGraphEstimationModels. All analyses were done on simulated and publicly available data. The Caenorhabditis elegans neural network data were retrieved from Chen et al. The Google Scholar MACN data were retrieved from https://github.com/kalhorghazal/Google-Scholar-Paper, as made available by Kalhor et al. The Saccharomyces cerevisiae RNA-seq data were retrieved from the ARCHS4 Zoo database.
